# Parental autonomy support and future education planning among Chinese senior high school students: a chain mediation model integrating self-determination theory and social cognitive theory

**DOI:** 10.3389/fpsyg.2025.1646811

**Published:** 2026-01-08

**Authors:** Guirong Liu, Mengyun Zhang, Xiaomei Zhang, Guizhou Yang, Feng Wang, Jinghuan Zhang, Xiuqin Teng, Yan Wang

**Affiliations:** 1 Department of Teacher and Education, Qilu Normal University, Jinan, China; 2 Department of Psychology, Shandong Normal University, Jinan, China; 3 Shandong Yiheng Institute of Psychology, Jinan, China

**Keywords:** academic self-efficacy, basic psychological needs satisfaction, future education planning, parental autonomy support, self-determination theory, social cognitive theory

## Abstract

**Introduction:**

Parental autonomy support (PAS) has been identified as a key factor in shaping adolescents’ educational development, yet little research has examined how PAS predicts students’ future educational planning (FEP) and whether this relationship is mediated by specific motivational mechanisms.

**Methods:**

Drawing on an integrative framework combining Self-Determination Theory (SDT) and Social Cognitive Theory (SCT), the present study investigated whether senior high school students’ basic psychological needs satisfaction (BPNS) and academic self-efficacy (ASE) sequentially mediate the relationship between PAS and FEP. A total of 1,591 Chinese senior high school students from three schools in Jinan, Shandong Province completed standardized measures of PAS, BPNS, ASE, and FEP.

**Results:**

Results showed that PAS significantly predicted students’ FEP. BPNS alone mediated the relationship between PAS and FEP, and BPNS and ASE jointly served as significant chain mediators.

**Discussion:**

These findings underscore the role of supportive parenting and internal motivational resources—namely, the satisfaction of basic psychological needs and efficacy beliefs—in facilitating adolescents’ engagement in future-oriented educational planning during high school. The study also advances theoretical understanding by integrating SDT and SCT to illuminate a hierarchical motivational pathway linking parenting practices to future planning outcomes.

## Introduction

Senior high school students, situated at a critical developmental transition toward adulthood, undergo marked changes in cognitive, social, and emotional domains (Jopling et al., 2024). During this period, they are expected not only to meet academic demands and prepare for higher education, but also to begin forming long-term goals and planning their educational futures. Future educational planning (FEP)—defined as adolescents’ capacity and intention to make informed, proactive decisions about their academic pathways—has thus become a key developmental task during this stage (Plasman, 2018).

In response to the growing emphasis on talent development in the 21st century, the General Office of the State Council (2019) issued the *Guidelines on Advancing the Reform of Talent Cultivation in Senior High Schools in the New Era*, which underscore the importance of career education and individualized developmental guidance at the high school level. The *Guidelines* further emphasize the active role of families in supporting students’ autonomy and decision-making regarding their FEP.

Despite this policy-level emphasis, challenges persist in translating these aims into meaningful student behaviors. At the micro level, the family—particularly parents—plays a central role in shaping adolescents’ educational attitudes and decision-making readiness (Norris, 2020). Among various parenting practices, parental autonomy support (PAS) has been identified as a key socializing factor that fosters adolescents’ intrinsic motivation and volitional functioning, both of which are critical for future-oriented planning (Chen T. et al., 2025; Chen C. et al., 2025).

Building upon Self-Determination Theory (SDT) (Ryan and Deci, 2017) and Social Cognitive Theory (SCT) (Bandura, 1986), the current study investigates how PAS contributes to students’ engagement in FEP through the sequential mediation of senior high school students’ basic psychological needs satisfaction (BPNS)—namely autonomy, competence, and relatedness—and academic self-efficacy (ASE). While BPNS captures the internalization of parental support, ASE reflects students’ confidence in their academic competence (Şimşek and Demir, 2012; Zheng, 2024). Together, these constructs form a theoretically grounded pathway explaining how supportive parenting facilitates long-term educational motivation and planning.

By adopting a chain mediation model, this study aims to advance our understanding of the motivational mechanisms through which PAS fosters adolescents’ FEP, providing evidence-based insights into how family-based support can promote students’ future-oriented motivation and behaviors.

### Parental autonomy support and future education planning

As one of the key areas of future planning, FEP refers to the process through which individuals actively contemplate and explore their educational development, formulate concrete plans, and take actions to achieve them (Seginer, 2019). It is considered a critical component of ego-identity formation during adolescence (Chen et al., 2015). This construct primarily consists of two dimensions: exploration, which involves making informed decisions based on extensive information gathering about educational pathways; and commitment, which refers to taking concrete actions to achieve educational goals (Zhang et al., 2006). Examples of FEP include choosing a desired university major, devising strategies for the National College Entrance Examination (Gaokao), and preparing for the educational requirements of future careers.

According to SDT, individuals possess an inherent tendency toward growth and self-actualization (Ryan and Deci, 2017). For senior high school students, proactively planning for future education represents a self-determined behavior, the extent of which is closely related to the autonomy-supportive qualities of their environment (Xu et al., 2019). Among environmental influences, the family plays a consequential role, with parents being the most immediate and influential figures in adolescents’ development. Parental behavior and the overall family climate are crucial to adolescents’ career development and realization of self-worth (Deng and Yang, 2022).

PAS refers to parenting behaviors that involve acknowledging and respecting children’s perspectives, encouraging independent decision-making, and providing meaningful information (Ryan and Deci, 2017; Grolnick and Ryan, 1989). Rooted in SDT, an autonomy-supportive environment is believed to foster positive behavioral outcomes (Ryan and Deci, 2017). In recent years, domestic research in China has also accumulated substantial empirical evidence underscoring the crucial role of PAS in adolescent development. For instance, Chen T. et al. (2025) and Chen C. et al. (2025), employing a longitudinal design, revealed a significant mutual promotion between PAS and adolescents’ academic engagement. Another study (Jiang et al., 2022) revealed that although parental autonomy support did not directly predict college students’ academic engagement, it indirectly promoted it through the serial mediation of career adaptability and career decision-making self-efficacy. This mechanism was also observed for teacher support and peer support, highlighting the fundamental nurturing role of PAS in individual development. Collectively, these studies indicate that within China’s highly competitive educational context, investigating PAS and its underlying psychological mechanisms is crucial for understanding and promoting positive adolescent development.

Empirical studies have demonstrated that PAS can stimulate adolescents’ proactive engagement in future planning by encouraging self-exploration, respecting their opinions and choices, and providing valuable guidance (Zeng et al., 2022). Specifically, when parents adopt autonomy-supportive practices, adolescents are more likely to actively explore different developmental possibilities, better integrate their interests and abilities with external opportunities, and formulate more concrete and feasible educational plans (Froiland, 2015). Moreover, parental understanding, respect, and support create a secure environment for exploration, enabling adolescents to face future choices with greater confidence, stronger planning motivation, and enhanced action capacity (Deng, 2024).

### Basic psychological needs satisfaction as a mediator

According to SDT, individuals are more likely to exhibit positive behavioral outcomes when the environment effectively supports the satisfaction of their basic psychological needs (Vansteenkiste et al., 2020). BPNS, a core construct of SDT, refers to the extent to which individuals experience fulfillment in autonomy, competence, and relatedness (Ryan and Deci, 2017).

Parents, as key figures within the family environment, are regarded as one of the most crucial external resources for fostering BPNS (Niemiec and Ryan, 2009). Specifically, PAS enhances needs satisfaction through multiple pathways: autonomy by respecting adolescents’ choices and supporting independent decision-making (Akram et al., 2022); relatedness by understanding and validating their thoughts and behaviors to nurture close parent–child relationships (Costa et al., 2019; van der Kaap-Deeder, 2021; Zhou et al., 2019; Inguglia et al., 2018); and competence by providing emotional support that builds confidence in their abilities and success potential (Germani and Palombi, 2022).

Empirical studies have consistently demonstrated that BPNS is strongly associated with future-oriented behaviors (Vansteenkiste et al., 2020). When these needs are adequately met, individuals are more likely to develop strong intrinsic motivation, a sense of agency, and greater engagement in exploratory behaviors—including future planning (Liu et al., 2023). In the educational context, such needs satisfaction has been found to enhance students’ motivation to explore educational paths, increase their investment in academic goals, and foster the formation of clearer, more feasible FEP (Manzano-Sánchez et al., 2021). Thus, BPNS plays a vital mediating role in the relationship between PAS and adolescents’ proactive planning for their educational futures.

### Academic self-efficacy as a mediator

In addition to BPNS, ASE represents another critical positive psychological cognitive process that may serve as a mediator between PAS and FEP. According to SCT, the influence of external environments on individual behavior operates indirectly through internal cognitive processes (Bandura, 1997; Schunk and Usher, 2012). Within the domain of FEP, PAS—as a significant environmental factor—not only directly affects adolescents’ planning behavior but may also exert indirect influence by shaping their psychological cognitions.

ASE refers to students’ confidence in their learning abilities and academic achievement; it is the belief in one’s capability to successfully complete academic tasks and attain educational goals (Honicke and Broadbent, 2016). Bandura posits that encouragement and verbal persuasion from significant others play an important role in enhancing self-efficacy (Bandura, 1986). Empirical research further supports this, showing that individuals who receive greater support from others tend to exhibit higher levels of ASE (Chen T. et al., 2025; Chen C. et al., 2025). When adolescents perceive autonomy support from their parents, such a supportive environment conveys external encouragement and trust, which directly strengthens their confidence and determination in the face of academic challenges, thereby enhancing their level of ASE (Deng, 2024).

Moreover, students with higher ASE tend to maintain a more positive attitude toward their future educational development. ASE influences how students confront functional and environmental challenges (Bassi et al., 2007), promotes the use of effective learning strategies, and predicts better educational outcomes (Bouffard-Bouchard et al., 1991; Schunk and Gunn, 1986; Ye, 2021). Through heightened ASE, students are more likely to pursue long-term ambitions, exhibit strong goal commitment, and maintain perseverance in the face of obstacles and setbacks (Bandura et al., 1999; Wright et al., 2012). Conversely, students with low ASE may lack both the motivation to set educational goals and the confidence to achieve them, thus hindering the formation and realization of future educational plans (Ryan and Deci, 2020). Therefore, ASE plays a significant mediating role in the relationship between PAS and senior high school students’ FEP.

### Association between basic psychological needs satisfaction and academic self-efficacy

Within the integrated framework of SDT and SCT, BPNS and ASE are hypothesized to not only independently mediate the PAS-FEP link but also to form a sequential mediation mechanism. The two theories offer complementary perspectives: SDT conceptualizes basic psychological needs as innate and universal motivators, while SCT posits that self-efficacy is an outcome-driven belief in one’s capabilities (Bandura, 1997). Given that self-efficacy is shaped by social experiences, the satisfaction of basic psychological needs can be viewed as a fundamental social antecedent in the formation of self-efficacy beliefs. Empirical evidence supports this linkage, indicating that BPNS serves as a precursor to self-efficacy beliefs in various educational settings (e.g., Basileo et al., 2024; Macakova and Wood, 2020). Thus, a supportive environment that fulfills psychological needs likely provides a foundational motivational substrate for the development of domain-specific self-efficacy.

### Theoretical integration and the chain mediation model

The core proposition of Self-Determination Theory (SDT)—that satisfying the three basic psychological needs for autonomy, competence, and relatedness is essential for promoting well-being, intrinsic motivation, and positive development—is considered to have cross-cultural universal applicability (Ryan and Deci, 2017). Early perspectives suggested that the concept of “autonomy” was primarily applicable to Western individualistic cultures, but extensive empirical research has refuted this view. Studies indicate that although the specific “ways” in which these needs are satisfied (e.g., which behaviors are perceived as autonomy-supportive) may vary across cultural contexts (Chirkov and Ryan, 2001), the needs themselves remain indispensable for positive individual development (Vansteenkiste et al., 2020). For example, in East Asian collectivist cultures, including China, consistent research has found that parental and teacher autonomy support significantly predicts students’ higher learning engagement, better academic achievement, and enhanced well-being by satisfying their basic psychological needs (Jiang et al., 2022; Zhou et al., 2019). Furthermore, recent research in Spanish physical education has shown that SDT-based training can effectively enhance teachers’ willingness to adopt autonomy-supportive teaching styles (Ocete et al., 2025). However, although the cross-cultural applicability of SDT is well-established, and despite initial attempts to integrate it with Social Cognitive Theory (SCT)—such as research exploring the impact of teaching styles on university students’ academic self-concept in Mexican physical education (Espinoza-Gutiérrez et al., 2024)—research that systematically integrates these two theoretical frameworks to elucidate the chain mediating motivational mechanism from familial environmental support to individuals’ long-term planning, particularly within the Chinese cultural context, remains scarce. This theoretical gap limits our in-depth understanding of how family environments shape students’ long-term developmental trajectories.

To address this research gap, the present study proposes a chain mediation model grounded in the aforementioned theoretical and empirical foundations, integrating SDT and SCT into a unified framework to explain the mechanism through which PAS influences FEP. SDT illuminates the satisfaction of basic psychological needs as a universal, intrinsic motivational foundation, whereas SCT elucidates the domain-specific cognitive beliefs formed thereon. Specifically, an environment that fulfills the needs for autonomy, competence, and relatedness—as emphasized by SDT—provides individuals with essential psychological security and intrinsic motivation, encouraging them to confront challenges and engage in exploration. This, in turn, increases the likelihood of accumulating successful experiences and building confidence in specific domains such as academics, thereby fostering higher self-efficacy—a core concept of SCT. Consequently, we hypothesize that basic psychological need satisfaction (BPNS) not only directly mediates the relationship between PAS and FEP but also serves as an antecedent to ASE. Together, these constructs form a chain pathway: “environmental support → psychological need satisfaction → domain-specific confidence → long-term planning.” The integrated model aims to demonstrate how supportive contexts are internalized through layered motivational processes to shape long-term, future-oriented outcomes.

In this model, PAS is the parental environmental factor, while BPNS, ASE, and FEP are all students’ internal perceptions and characteristics. This distinction clarifies the hierarchical pathway from external support to internal motivational processes and ultimately to future-oriented behavior.

### Present study

**Figure 1:**
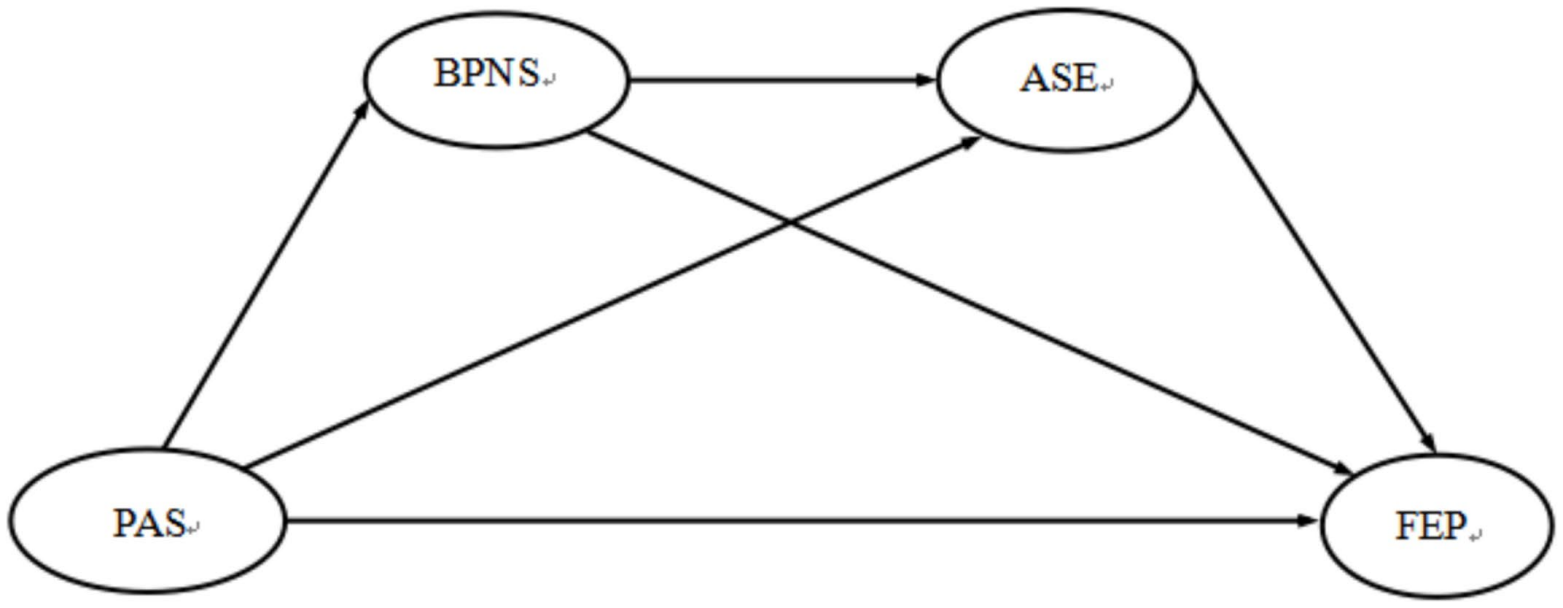
The hypothesized chain mediation model of basic psychological need satisfaction and academic self-efficacy between parental autonomy support and future educational planning. PAS, parental autonomy support; BPNS, basic psychological needs satisfaction; ASE, academic self-efficacy; FEP, future educational planning.

Based on the integrative model, the present study examines the motivational mechanisms linking PAS to FEP among Chinese senior high school students (see Figure 1). The following hypotheses are proposed:

*Hypothesis 1:* PAS positively predicts senior high school students' FEP.

*Hypothesis 2:* BPNS mediates the relationship between PAS and FEP.

*Hypothesis 3:* ASE mediates the relationship between PAS and FEP.

*Hypothesis 4:* BPNS and ASE sequentially mediate the relationship between PAS and FEP.

## Method

### Participants

The sample (*N* = 1,591) was drawn from three public senior high schools located in Jinan City, Shandong Province, China. A convenience sampling method was employed, with the selected schools covering diverse geographical locations (including both urban and suburban areas). The participants included 794 males (49.906%) and 797 females (50.094%), comprising 582 eleventh-grade (i.e., Senior 1), 512 twelfth-grade (i.e., Senior 2), and 497 thirteenth-grade (i.e., Senior 3). The average age of the participants was 16.12 years (SD = 0.91) (see Table 1).

**Table 1:** Sample description.

Grade	Male	Female	Total	M_age_
Eleventh	289	293	582	15.20 ± 0.42
Twelfth	243	269	512	16.15 ± 0.43
Thirteenth	262	235	497	17.16 ± 0.43
Total	794	797	1,591	16.12 ± 0.91

Information regarding the participants’ family socioeconomic background is presented in Table 2. In terms of parental education levels, 47.83% of fathers and 44.69% of mothers had attained a college/university degree or higher. Regarding occupational distribution, the majority of parents were categorized as “workers, private business owners, or self-employed” accounting for 46.51% of fathers and 37.96% of mothers. The next most common categories were “middle- or senior-level positions in enterprises, public institutions, or government” for fathers (19.48%) and “commercial or service industry workers” for mothers (22.50%). A smaller proportion of fathers (3.83%) and mothers (11.31%) were classified as farmers or had no stable occupation. In terms of total monthly household income, the majority of families (41.73%) reported income in the range of 9,000 to 20,000 RMB, followed by 22.44% in the 6,000 to 9,000 RMB range, and 21.43% reporting more than 20,000 RMB.

**Table 2:** SES description.

	Parental education					Parental occupation				Family monthly income		
Category	Father		Mother		Category	Father		Mother				
	Number	Percentage	Number	Percentage		Number	Percentage	Number	Percentage	Category	Number	Percentage
Elementary school or below	41	2.58	62	3.90	Farmer or unemployed	61	3.83	180	11.31	Below 3,000 yuan	46	2.89
Junior high school	295	18.54	342	21.50	Worker, private business owner, or self-employed	740	46.51	604	37.96	3,000–6,000 yuan	183	11.50
High school or technical secondary school	494	31.05	476	29.92	Commercial or service worker	233	14.64	358	22.50	6,000–9,000 yuan	357	22.44
Junior college/undergraduate	682	42.87	645	40.54	Government official; ordinary military personnel; teacher, doctor, researcher; professional technician	247	15.52	234	14.71	9,000–20,000 yuan	664	41.73
Graduate student	79	4.97	66	4.15	Corporate manager; government clerk; senior executive of public institution; military officer	310	19.48	215	13.51	Above 20,000 yuan	341	21.43

### Instruments

#### Future educational planning

FEP among senior high school students was measured using the *Adolescent Future Orientation Subscale* (Zhang et al., 2006). This section consists of 7 items, divided into two dimensions: exploration and commitment. Responses were rated on a 5-point Likert scale, with higher average scores indicating a higher level of FEP among high school students. In the current study, the construct validity of FEP (e.g., “Do you often think about or plan your studies and the education you want to receive in the future?”) was found to be good, with fit indices as follows: *χ^2^*/df = 2.651, RMSEA = 0.032, SRMR = 0.011, CFI = 0.995, and TLI = 0.988. The Cronbach’s *α* for the scale was 0.837, indicating a good internal consistency. All standardized factor loadings were significant and ranged from 0.53 to 0.74, demonstrating strong item representation.

#### Parental autonomy support

PAS was measured using the revised Parental Autonomy Support Scale (Wang et al., 2007). The scale consists of 8 items across two dimensions: choice making (e.g., “Whenever possible, my parents allow me to make my own choices”) and opinion exchange (e.g., “When I encounter problems, my parents listen to my opinions and viewpoints”). Responses were rated on a 5-point Likert scale ranging from “strongly disagree” to “strongly agree” Higher average scores indicate a greater perceived level of PAS. The scale demonstrated good construct validity (*χ^2^*/df = 3.381, RMSEA = 0.039, SRMR = 0.012, CFI = 0.994, TLI = 0.987), and the Cronbach’s *α* was 0.942, indicating excellent internal consistency. All standardized factor loadings were significant and ranged from 0.75 to 0.90, demonstrating strong item representation.

#### Basic psychological needs satisfaction

BPNS was measured using the Basic Psychological Need Satisfaction Scale (Van der Kaap-Deeder et al., 2020). This scale includes 12 items divided into three dimensions: 4 items assess autonomy need satisfaction (e.g., “I feel a sense of freedom to choose when doing things”), 4 items assess competence need satisfaction (e.g., “I feel confident that I can do things well”), and 4 items assess relatedness need satisfaction (e.g., “I feel that the people I care about also care about me”). Responses were rated on a 5-point Likert scale ranging from “not at all true” to “completely true,” with higher average scores indicating a higher level of BPNS. The average scores for autonomy, competence, and relatedness satisfaction were calculated separately and used as indicators of BPNS in the SEM analysis. The scale was translated and adapted through multiple rounds of forward and back-translation by a panel consisting of two psychology professors and six graduate students specializing in psychology and English, ensuring cross-cultural appropriateness. The results indicated a well-fitting structural model with no items removed. In the present study, the scale demonstrated good construct validity (*χ^2^*/df = 2.775, RMSEA = 0.033, SRMR = 0.024, CFI = 0.982, TLI = 0.975), and the Cronbach’s *α* was 0.905. All standardized factor loadings were significant and ranged from 0.60 to 0.82, demonstrating strong item representation.

#### Academic self-efficacy

ASE was assessed using the Academic Self-Efficacy subscale from the Patterns of Adaptive Learning Scales (Jia et al., 2020; Midgley et al., 2000), which includes 5 items (e.g., “I believe I can master what the teacher teaches in class”). Responses were rated on a 5-point Likert scale ranging from “strongly disagree” to “strongly agree” Higher average scores indicate greater confidence among high school students in overcoming academic difficulties or setbacks, reflecting higher levels of ASE. In the present study, the scale demonstrated good construct validity (*χ^2^*/df = 1.733, RMSEA = 0.021, SRMR = 0.007, CFI = 0.999, TLI = 0.997), and the Cronbach’s α was 0.919, indicating strong internal consistency. All item loadings were significant and ranged from 0.74 to 0.90, demonstrating strong item representation.

#### Family socioeconomic status (SES)

In this study, family socioeconomic status (SES) was assessed using three indicators: parental education, parental occupation, and monthly household income. Following previous research, parental occupation and education levels were categorized into five and six levels, respectively (Callahan and Eyberg, 2010), and were coded in ascending order. Parental occupation was scored from 1 to 5, with higher scores indicating a higher level of occupational expertise or professional skill. Parental education was scored from 1 to 6, with higher scores representing higher educational attainment. Monthly household income was scored on a 13-point scale, ranging from 1 (less than 1,000 RMB) to 13 (30,000 RMB or above), with higher scores indicating greater income.

A composite SES score was computed following standard procedures used in previous studies (Delore et al., 2024); detailed computation methods are provided in Appendix S6.

### Procedure

This study received ethical approval from the University Research Ethics Review Committee at Qilu Normal University. Prior to participation, both parental and student informed consent was obtained. Data collection was conducted collectively at the class level. Two graduate students majoring in psychology served as examiners, clearly explaining the research purpose and confidentiality principles to participants. Students were given approximately 20 min to complete the questionnaire, which included items on demographic information, PAS, BPNS, ASE, and FEP. Upon completion, the examiners reviewed and collected the questionnaires and provided small gifts to participants as a token of appreciation.

### Data analysis

As a cross-sectional study, this study adhered to the STROBE guidelines for observational studies (Von Elm et al., 2008). To begin, descriptive statistical analyses were performed using SPSS 27.0 following established best-practice guidelines (Ibáñez-López et al., 2024), a common method bias test was conducted. Furthermore, we calculated the Intraclass Correlation Coefficients (ICCs) to quantify the proportion of variance explained at the classroom level, given the nested structure of the data (students within classes). Thereafter, mediation effects within the research model were tested using Mplus 8.3. All statistical analyses employed the bootstrap method with 5,000 resamples to compute the 95% confidence intervals of the indirect effects (Muthen and Muthen, 2017).

### Common method bias test

In this study, all key variables were collected through participants’ self-reports. To minimize the potential impact of CMB, procedural and statistical controls were employed. Procedurally, participants completed the survey anonymously, and items that could cause ambiguity were revised or clarified. Statistically, Harman’s single-factor test was used (Harman, 1960). The results showed that five factors had eigenvalues greater than 1, and the first factor accounted for 33.37% of the variance. Since the explained variance by the first common factor was below the critical threshold of 40%, it indicates that no significant CMB was present in this study.

## Results

### Descriptive statistics, correlation, and intraclass correlation

The study found significant positive correlations among PAS, ASE, BPNS, and FEP, providing preliminary support for the theoretical hypotheses. Additionally, gender differences were observed in PAS and ASE (see Table 3). Furthermore, Intraclass Correlation Coefficients (ICCs) were calculated and a multi-level linear modeling analysis was conducted. The results showed that all ICC values were below 4%, indicating minimal explainable variance at the classroom level (see Appendix S1). Moreover, the coefficient estimates of HLM for key pathways were consistent in direction with the Mplus analysis, confirming the robustness of the research findings (see Appendix S2).

**Table 3:** *M*, SDs, and correlations among study variables.

Variable	1	2	3	4	5	6
1 Gender	–					
2 SES	0.05^***^	–				
3 PAS	0.07^***^	0.12^***^	–			
4 BPNS	0.01^***^	0.11^***^	0.39^***^	–		
5 ASE	0.17^***^	0.13^***^	0.28^***^	0.60^***^	–	
6 FEP	−0.01^***^	0.19^***^	0.26^***^	0.41^***^	0.37^***^	–
*M*	0.50^***^	0.00^***^	3.87^***^	3.75^***^	3.64^***^	3.60^***^
*SD*	0.50^***^	1.00^***^	0.92^***^	0.71^***^	0.92^***^	0.68^***^

^*^p < 0.05, ^**^p < 0.01, ^***^p < 0.001, *N* = 1,591. SES, family socioeconomic status; PAS, parental autonomy support; BPNS, basic psychological needs satisfaction; ASE, academic self-efficacy; FEP, future educational planning. Girls were coded as “0” and boys were coded as “1”.

### Measurement model

As shown in Table 3, all variables measured in this study demonstrated good internal consistency, with Cronbach’s *α* coefficients all exceeding 0.80. To address the construct validity of the five latent constructs, a confirmatory factor analysis (CFA) was conducted following current reporting standards for structural equation modeling (Kline, 2023). The results indicated a good model fit to the data, with all indices meeting recommended thresholds: *χ^2^*/df = 3.86, CFI = 0.949, TLI = 0.938, SRMR = 0.008, RMSEA = 0.042. All factor loadings were statistically significant and exceeded 0.60. The composite reliability (CR) values were all above 0.67, and the average variance extracted (AVE) values were all greater than 0.50, indicating adequate convergent validity of the model (see Appendix S3). Furthermore, to examine discriminant validity, the heterotrait-monotrait ratio (HTMT) was calculated. As shown in Appendix S4, all HTMT values ranged from 0.281 to 0.592, all well below the conservative threshold of 0.85 (Henseler et al., 2015), providing strong evidence for the discriminant validity of the measurement model.

To ensure that the measurement model held equivalent meaning across different demographic groups, we tested for measurement invariance across gender and grade levels. The results supported full scalar invariance for both grouping variables, as the changes in comparative fit index (ΔCFI) were all less than 0.010 and the changes in RMSEA (ΔRMSEA) were all less than 0.015 when comparing the scalar model with the configural and metric models. This indicates that the factor structure, factor loadings, and item intercepts were equivalent across groups, thus justifying meaningful comparisons of structural paths and latent means across different genders and grade levels. Detailed fit indices are provided in Appendix S6.

### Structural model

The structural equation modeling analysis indicated that the model fit indices reached acceptable levels: *χ^2^*/df = 4.98, CFI = 0.979, TLI = 0.969, SRMR = 0.032, and RMSEA = 0.050.

In the mediation analysis, gender, age, and family SES were included as control variables. The results for control variables showed that both SES (*β* = 0.18, *p* < 0.001) and age (*β* = 0.10, *p* < 0.001) had significant positive effects on FEP, while gender (*β* = −0.07, *p* < 0.01) showed a significant negative effect, indicating that male students reported significantly lower levels of FEP than female students.

**Figure 2:**
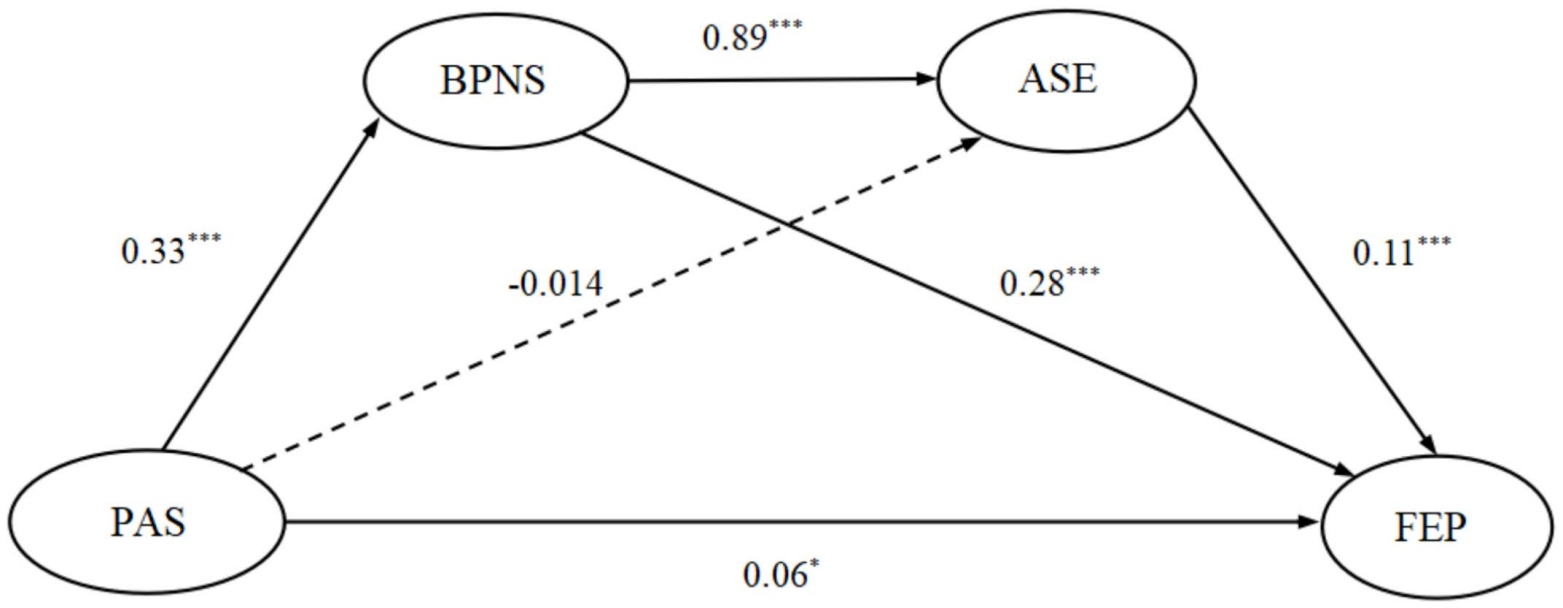
The chain mediation mechanism of basic psychological need satisfaction and academic self-efficacy between parental autonomy support and future educational planning. PAS, parental autonomy support; BPNS, basic psychological needs satisfaction; ASE, academic self-efficacy; FEP, future educational planning. Solid lines indicate significant relationships, and dashed lines indicate non-significant relationships.

As shown in Table 4 and Figure 2, regarding the hypothesized paths, PAS significantly and positively predicted FEP (*β* = 0.18, *p* < 0.001). After including the mediators and control variables, the direct effect of PAS on FEP remained significant (*β* = 0.06, *p* < 0.05, 95% CI [0.02, 0.11]), thus supporting Hypothesis 1.

**Table 4:** The test of mediating effects.

Path	Estimate	SE	Effect size	95% CI	
				Lower	Upper
**PAS → FEP**	0.06^*^	0.03	33.33%	0.02	0.11
**PAS → BPNS → FEP**	0.09^***^	0.02	50.00%	0.07	0.12
PAS → ASE → FEP	−0.002	0.03	–	−0.07	0.003
**PAS → BPNS → ASE → FEP**	0.03^***^	0.08	16.67%	0.02	0.04
**Total effect**	0.18^***^	0.02	–	0.26	0.35

^*^p < 0.05, ^**^p < 0.01, ^***^p < 0.001. The pathways in bold are that the upper and lower limits of the 95% CI do not include zero. CI, confidence interval; PAS, parental autonomy support; BPNS, basic psychological needs satisfaction; ASE, academic self-efficacy; FEP, future educational planning.

For mediation effects, PAS positively predicted BPNS (*β* = 0.33, *p* < 0.001), and BPNS positively predicted FEP (*β* = 0.28, *p* < 0.001). The mediating effect of BPNS between PAS and FEP was significant (estimate = 0.09, 95% CI [0.07, 0.12]), supporting Hypothesis 2. However, PAS did not significantly predict ASE (*β* = −0.014, *p >* 0.05), although ASE positively predicted FEP (*β* = 0.11, *p* < 0.001), and consequently, the mediating effect of ASE was not significant (estimate = −0.002, 95% CI [−0.07, 0.003]). Thus Hypothesis 3 was not supported. Furthermore, BPNS positively predicted ASE (*β* = 0.89, *p* < 0.001), and the chain mediation effect through BPNS and ASE was significant (estimate = 0.03, 95% CI [0.02, 0.04]), supporting Hypothesis 4.

The squared multiple correlations (R^2^) indicated that the model explained 25.6% of the variance in BPNS, 45.1% in ASE, and 31.4% in FEP. Following Cohen’s (1992) guidelines, the explanatory power for BPNS and FEP can be considered large, as is the case for ASE.

## Discussion

Guided by an integrative framework of SDT and SCT, this study investigated the mechanisms linking PAS to FEP among Chinese senior high school students. The results revealed three primary findings. First, PAS demonstrated a significant direct effect on FEP, establishing a robust foundational relationship. Second, BPNS served as a pivotal mediator, delineating the intrinsic pathway through which autonomy support operates. Third, and most notably, a significant sequential mediation pathway was identified, connecting PAS to FEP via BPNS and then ASE. This chain mediation elucidates a hierarchical motivational sequence from global psychological needs to domain-specific efficacy beliefs. Collectively, these findings illustrate how PAS shapes adolescent future orientation through multi-layered motivational processes.

### Parental autonomy support—future educational planning link

A primary and central finding of this study is that PAS exerts a significant positive predictive effect on senior high school students’ FEP, thereby supporting Hypothesis 1. Notably, even after accounting for the mediating roles of BPNS and ASE, the direct effect of PAS on FEP remained significant.

This result underscores that the general climate of autonomy and volition fostered by parents not only functions indirectly by nurturing adolescents’ internal psychological resources but also exerts an indispensable and independent influence on their future-oriented planning behaviors. This pattern suggests that the open communication and respect inherent in PAS may empower adolescents directly during the processes of future exploration and commitment, above and beyond the internal pathways through BPNS and ASE.

This robust direct effect provides strong support for SDT (Ryan and Deci, 2017), which posits that autonomy-supportive environments facilitate self-determined behavior. Within the Chinese cultural context—often characterized by collectivism and parental authority (Chao, 1994)—this finding is particularly noteworthy. It demonstrates that even in a cultural environment where familial obedience may be emphasized, providing autonomy support can effectively enhance adolescents’ initiative in planning their educational futures, thereby attesting to the cross-cultural robustness of SDT.

### The mediating role of basic psychological needs satisfaction

The present study identifies senior high school students’ BPNS as a central psychological mechanism through which PAS promotes adolescents’ FEP. Conceptualized not as a static psychological state but as a dynamic motivational process, BPNS represents the internalization of contextual support (i.e., PAS) by the students. It functions as a key conduit, transforming external parental autonomy support into the internal psychological resources of the adolescent, which are necessary for intentional, future-oriented behavior.

This finding aligns with the core proposition of SDT (Ryan and Deci, 2017), which posits that fulfilling the needs for autonomy, competence, and relatedness facilitates the internalization of values and the development of sustained self-regulation. In the context of parenting, autonomy support promotes these needs through multiple complementary pathways: it nurtures autonomy by encouraging volitional decision-making; fosters relatedness through emotional affirmation and understanding; and builds competence by providing positive reinforcement that bolsters confidence in one’s abilities (Costa et al., 2019; Germani and Palombi, 2022).

The significant indirect effect underscores BPNS as a central motivational mechanism that channels PAS into adolescents’ psychological readiness and behavioral engagement with long-term educational planning. This finding reinforces the notion that FEP is not merely a cognitive or strategic task, but one that is fundamentally rooted in the quality of motivational experience. Within this framework, BPNS serves as the foundational pathway through which environmental support is internalized and transformed into future-directed action.

### The mediating role of academic self-efficacy

Contrary to Hypothesis 3, PAS did not directly predict ASE, nor did ASE independently mediate the relationship between PAS and FEP. This non-significant finding, however, is theoretically clarifying. The absence of a direct link can be primarily attributed to a conceptual and temporal misalignment between the constructs: ASE pertains to confidence in mastering *current* academic tasks, whereas FEP involves *future-oriented* processes like setting long-term goals and exploring educational trajectories. This fundamental mismatch explains why ASE functions more effectively as a subsequent link in the motivational chain rather than as an independent mediator. The strong path from BPNS to ASE confirms this sequential relationship, positioning SDT’s core construct as a critical antecedent to SCT’s core construct.

Furthermore, within the Chinese educational context, ASE is heavily shaped by performance-based experiences—such as academic success and evaluative feedback—which may exert a more immediate influence on efficacy beliefs than generalized parental support (Bandura, 2001; Chen T. et al., 2025; Chen C. et al., 2025). In this environment, PAS operates not by directly competing with these potent external indicators, but upstream by providing a foundational supportive environment that cultivates the inner resources (i.e., BPNS) necessary for developing robust efficacy beliefs, a view supported by other empirical findings (Tazouti and Jarlégan, 2019).

Finally, this nuanced result invites consideration of potential boundary conditions. Following the motivational intervention perspective of Franco et al. (2025), the role of ASE might depend on individuals’ motivational readiness. Therefore, future research should examine whether motivational variables moderate this pathway, thereby deepening our understanding of the complex mechanisms at play.

### The sequential mediating roles of basic psychological needs satisfaction and academic self-efficacy

The significant sequential mediation effect supports Hypothesis 4, providing the most comprehensive account of how PAS influences FEP through BPNS and ASE. This model validates our theoretical framework by demonstrating a motivational sequence from the broad foundations of SDT to the domain-specific mechanisms of SCT.

Specifically, the robust “BPNS → ASE” path substantiates this theoretical link. The strength of this relationship is a substantive finding, and multicollinearity diagnostics (all VIFs < 3) confirmed stable parameter estimates. This path empirically validates that basic psychological need satisfaction is a powerful, foundational antecedent to academic self-efficacy (Basileo et al., 2024). The fulfillment of autonomy, competence, and relatedness needs provides the internal resources that enable students to experience volition, build influence over their environment, and feel supported—thereby boosting their academic confidence (Ryan and Deci, 2017). In this process, BPNS serves as the essential psychological substrate for ASE, which in turn acts as a motivational catalyst for future planning (Zhen et al., 2017).

Our dimensional analysis offers a concrete illustration of this sequence, revealing a salient “autonomy support-commitment” pathway. We found that the parental practice of “choice making” was most strongly translated into adolescents’ “commitment” to their educational plans. This pattern grounds our model in reality, underscoring that granting decision-making power is directly linked to the willingness to invest effort into future goals.

In summary, by examining the mediating roles of BPNS and ASE, this study deepens our understanding of the core relationship between PAS and adolescents’ FEP. Our findings go beyond demonstrating that PAS predicts FEP; they illuminate the sequential internal processes through which this influence occurs. Supportive parenting first fosters a sense of BPNS within the adolescent. This enriched inner state, in turn, fertilizes the ground for cultivating domain-specific academic confidence, ultimately forming a complete motivational pathway from contextual support to intrinsic needs, to domain-specific efficacy, and finally to future-oriented behavior.

### Strengths, limitations, and future directions

The study presents a theoretically grounded model of adolescents’ FEP by integrating SDT and SCT, illustrating a hierarchical motivational pathway, and supporting SDT’ cross-cultural applicability in China. Despite these contributions, several limitations should be acknowledged.

First, the cross-sectional design precludes causal inferences and cannot model developmental dynamics. While longitudinal designs are a natural recommendation, repeated testing can introduce practice effects and participant fatigue (Zyga et al., 2022). Future studies could therefore adopt cross-sequential designs, which combine longitudinal follow-up with cross-sectional sampling, to more robustly capture the evolving relationships between parenting and adolescent planning while mitigating the drawbacks of purely longitudinal approaches (Ahn et al., 2022; Gao et al., 2022).

Second, although self-reports were appropriate for capturing subjective perceptions, potential biases (e.g., social desirability) cannot be ruled out. Employing multi-informant reports or integrating qualitative methods would strengthen measurement validity through triangulation (Bi et al., 2022; Liu et al., 2013; Rohner and Lansford, 2017).

Third, the non-significant mediating role of ASE invites further investigation. We propose that a temporal misalignment may be a key factor: ASE concerns *current* academic capabilities, whereas FEP involves *future-oriented* planning. This suggests that the temporal congruence between mediators and outcomes may be more critical than domain similarity for explanatory power. Future studies should directly examine how temporal orientation moderates motivational pathways. Furthermore, exploring boundary conditions is essential. Variables such as age, gender, and family socioeconomic status SES—which correlated with all key variables—may moderate these pathways. For instance, future research could test whether SES strengthens or weakens the link between PAS and ASE, offering a more nuanced understanding of how family context shapes motivational processes.

Finally, the generalizability of our findings should be tested across China’s diverse socioeconomic and regional contexts. Participants were recruited from one city, and factors like varying educational resources, family concepts, and academic pressure in other regions may influence how PAS is perceived and operates. Future studies should expand sampling to determine whether macro-level sociocultural factors moderate the identified mechanisms.

### Practical implications

This study elucidates the motivational sequence from PAS to FEP, providing an empirical basis for translating national educational policy into family practice. Our findings affirm the emphasis on familial roles outlined in the *Guidelines on Promoting the Reform of Educational Methods in Regular Senior Secondary Schools in the New Era* (General Office of the State Council, 2019), by identifying PAS as a key mechanism and demonstrating its operation through the cultivation of adolescents’ BPNS and ASE.

Consequently, the most effective parental role shifts from direct academic intervention to the creation of an autonomy-supportive environment. Parents can be guided to implement this approach through several core practices: establishing regular, non-judgmental dialogues to explore their child’s academic interests and future aspirations; offering guided choices with clear rationale in key decisions to foster competence in decision-making; collaboratively breaking down long-term educational objectives into manageable steps to build a sense of control; and responding to setbacks with empathetic support that frames challenges as learning opportunities. These targeted practices are precisely designed to nurture the intrinsic motivational resources (BPNS and ASE) that our model identifies as the engine for proactive FEP.

Therefore, policy initiatives aimed at enhancing student development should explicitly incorporate strategies to empower parents in this role. This entails moving beyond general encouragement to provide structured support through evidence-based parent education programs focused on autonomy-supportive skills, robust home-school collaboration mechanisms, and public resources that recast the parental identity from “outcome controller” to “autonomy facilitator.” Evidence-based interventions such as *The Incredible Years* and *Triple P* (Leijten et al., 2018; Baumann et al., 2015) offer valuable, culturally-adaptable models for this purpose. In this way, educational policy can strategically leverage the family ecosystem to cultivate a generation of students capable of self-determined and proactive planning. This approach is especially valuable in the Chinese context, providing a pathway for parents to support autonomy within a culture that places a strong emphasis on academic achievement.

## Conclusion

This study sheds light on how PAS shapes adolescents’ FEP through the sequential mediation of BPNS and ASE, highlighting the central role of basic psychological needs in fostering future-oriented motivation. The non-significant independent effect of ASE reveals the importance of conceptual and temporal alignment in efficacy-based models. Theoretically, the findings contribute to the integration of SDT and SCT by identifying a hierarchical motivational pathway from contextual support to long-term educational planning. Practically, they underscore the value of autonomy-supportive parenting in promoting adolescents’ engagement in future educational decision-making within the Chinese cultural context.

## Data Availability

The raw data supporting the conclusions of this article will be made available by the authors, without undue reservation.
