# Supplementary material for: Parental autonomy support and future education planning among Chinese senior high school students: a chain mediation model integrating self-determination theory and social cognitive theory
Source: Front Psychol. 2026 Jan 8;16:1646811. doi: 10.3389/fpsyg.2025.1646811 (PMC12823887; doi:10.3389/fpsyg.2025.1646811)
Supplement: Supplementary file 1 [file Table_1.DOCX]

**Online Supplemental Materials for**

**Parental Autonomy Support and Future Education Planning among Chinese Senior High School Students: A Chain Mediation Model Integrating Self-Determination Theory and Social Cognitive Theory**

**Authors’ note:**

We developed these materials to provide additional technical information and to keep the main manuscript from becoming needlessly long.

[Appendix S1 2](#_Toc16469)

[Appendix S2 3](#_Toc14066)

[Appendix S3 5](#_Toc2312)

[Appendix S4 6](#_Toc16495)

[Appendix S5 7](#_Toc25330)

[Formula and Statistical Principles for SES 8](#_Toc27703)

Appendix 1

## Appendix S1

Intraclass Correlation Coefficients (ICCs) for Key Study Variables

| Variable | ICC | Variance at Classroom Level | Variance at Individual Level |
| --- | --- | --- | --- |
| FEP | 2.25% | 2.25% | 97.75% |
| BPNS | 0.27% | 0.27% | 99.73% |
| ASE | 3.33% | 3.33% | 96.67% |

Appendix 2

## Appendix S2

Comparison of Mplus and HLM Results for Key Paths

| Tested Path | Analytical Method | Effect | Significance |
| --- | --- | --- | --- |
| **Direct Effect** |  |  |  |
| PAS→FEP | Mplus | 0.06 | ^*^ |
| PAS→FEP | HLM | 0.080 | ^***^ |
| **BPNS as Mediation** |  |  |  |
| PAS→BPNS→FEP | Mplus | 0.09 | ^***^ |
| PAS→BPNS→FEP | HLM | 0.072 | ^***^ |
| **ASE as Mediation** |  |  |  |
| PAS→ASE→FEP | Mplus | -0.002 | n.s. |
| PAS→ASE→FEP | HLM | 0.037 | ^***^ |
| **Chain Mediation** |  |  |  |
| PAS→BPNS→ASE→FEP | Mplus | 0.03 | ^***^ |
| PAS→BPNS→ASE→FEP | HLM | 0.030 | ^***^ |

*Note: n.s. = not significant; p^*^<0.05, ^**^p<0.01, ^***^p<0.001.

Appendix 3

## Appendix S3

Results of Confirmatory Factor Analysis

| Latent Variable | Indicator | Standardized Loading | CR | AVE |
| --- | --- | --- | --- | --- |
| PAS | Choice Making | 0.718 | 0.670 | 0.503 |
|  | Opinion Exchange | 0.700 |  |  |
| BPNS | Autonomy | 0.797 | 0.823 | 0.613 |
|  | Competence | 0.622 |  |  |
|  | Relatedness | 0.904 |  |  |
| ASE | Item1 | 0.860 | 0.920 | 0.696 |
|  | Item2 | 0.832 |  |  |
|  | Item3 | 0.748 |  |  |
|  | Item4 | 0.876 |  |  |
|  | Item5 | 0.850 |  |  |
| FEP | exploration | 0.713 | 0.777 | 0.638 |
|  | commitment | 0.876 |  |  |

Note: PAS = Parental Autonomy Support; BPNS = Basic Psychological Needs Satisfaction; ASE = Academic Self-Efficacy; FEP = Future Educational Planning.

Appendix 4

## Appendix S4

Heterotrait-Monotrait (HTMT) Ratio Matrix for Discriminant Validity

| Latent Variable | PAS | BPNS | ASE | FEP |
| --- | --- | --- | --- | --- |
| PAS | - |  |  |  |
| BPNS | 0.432 | - |  |  |
| ASE | 0.281 | 0.592 | - |  |
| FEP | 0.319 | 0.535 | 0.493 | - |

Note: PAS = Parental Autonomy Support; BPNS = Basic Psychological Needs Satisfaction; ASE = Academic Self-Efficacy; FEP = Future Educational Planning.

Appendix 5

## Appendix S5

Full Results for Measurement Invariance Tests

| Model | *χ*^2^ | *df* | CFI | TLI | RMSEA [90%CI] | ΔCFI | ΔRMSEA |
| --- | --- | --- | --- | --- | --- | --- | --- |
| ***Gender*** |  |  |  |  |  |  |  |
| M1: Configural Invariance | 355.337 | 96 | 0.974 | 0.964 | 0.058[0.052, 0.065] | - | - |
| M2: Metric Invariance | 373.572 | 104 | 0.973 | 0.965 | 0.057[0.051, 0.063] | -0.001 | -0.001 |
| M3: Scalar Invariance | 391.917 | 110 | 0.971 | 0.966 | 0.057[0.051, 0.063] | -0.002 | 0.000 |
| ***Grade*** |  |  |  |  |  |  |  |
| M1: Configural Invariance | 488.067 | 152 | 0.967 | 0.957 | 0.065[0.058, 0.071] | - | - |
| M2: Metric Invariance | 515.795 | 168 | 0.966 | 0.959 | 0.062[0.056, 0.069] | -0.001 | -0.003 |
| M3: Scalar Invariance | 534.143 | 176 | 0.965 | 0.960 | 0.062[0.056, 0.068] | -0.001 | 0.000 |

Note: The changes in fit indices (ΔCFI and ΔRMSEA) support the establishment of scalar invariance for both gender and grade.

Appendix 6

## Formula and Statistical Principles for SES

Based on previous methodology for computing family SES (Delore et al., 2024), and considering the differing score ranges of the three indicators, each variable was first standardized into z-scores. Principal component analysis was then conducted on these standardized variables, and an SES composite score was computed for each participant using the following formula: Family SES = (*β*_1_ ×Zfather’s occupation + *β*_2_×Zmother’s occupation + *β*_3_ ×Zfather’s education + *β*_4_ ×Zmother’s education + *β*_5_ ×Zmonthly househoud income)/εf. Higher composite scores indicate a higher level of family socioeconomic status. In this study, the mean SES score of participants was 0.000 ± 1.000 (M ± SD), with a range from -2.58 to 2.37
